# Regionwide and Nationwide Floristic Richness Reveal Vascular Plant Diversity in Central Asia

**DOI:** 10.3390/plants13162275

**Published:** 2024-08-15

**Authors:** Suliya Ma, Wenjun Li, Komiljon Sh. Tojibaev, Orzimat Turginov, Weikang Yang, Keping Ma

**Affiliations:** 1 State Key Laboratory of Desert and Oasis Ecology, Key Laboratory of Ecological Safety and Sustainable Development in Arid Lands, Xinjiang Institute of Ecology and Geography, Chinese Academy of Sciences, No.818 South Beijing Road, Urumqi 830011, China; masuliya@163.com (S.M.); yangwk@ms.xjb.ac.cn (W.Y.); kpma@ibcas.ac.cn (K.M.); 2 Inner Mongolia University of Technology, No.49 Ai Min Road, Hohhot 010051, China; 3 Xinjiang Key Lab of Conservation and Utilization of Plant Gene Resources, No.818 South Beijing Road, Urumqi 830011, China; 4 State Key Laboratory of Vegetation and Environmental Change, Institute of Botany, Chinese Academy of Sciences, No.20 Nanxincun, Xiangshan, Beijing 100093, China; 5 Institute of Botany, Uzbekistan Academy of Sciences, No.32 Durmon Yuli Street, Tashkent 100125, Uzbekistan; orzimat@mail.ru; 6 College of Resources and Environment, University of Chinese Academy of Sciences, No.19(A) Yuquan Road, Shijingshan District, Beijing 100049, China

**Keywords:** Central Asia, vascular plant, floristic richness, endemic, regional

## Abstract

Central Asia (CA) is located in the interior of the Eurasian continent and consists of five countries—Kazakhstan, Kyrgyzstan, Tajikistan, Turkmenistan, and Uzbekistan. It contains the largest concentration of temperate deserts and mountains of CA biodiversity hotspots. However, regionwide floristic diversity is sorely lacking, and nationwide floristic diversity is seriously outdated in this region. Using the data collected by the Mapping Asia Plants (MAP) project, we describe and analyze the diverse floristic characteristics of plant diversity in CA at both the regional and national levels, including the dominant families and genera, endemic taxa, and floristic similarity. The results allow the compilation of a new checklist of vascular plants in CA, including 9643 taxa (1198 genera within 139 families) and 3409 endemic taxa (414 genera in 66 families). We confirm that there are 5695, 4036, 4542, 3005, and 4222 species of vascular plants within the CA countries, of which 532, 326, 505, 175, and 301 species are endemic taxa in Kazakhstan, Kyrgyzstan, Tajikistan, Turkmenistan, and Uzbekistan, respectively. The region’s biodiversity is notable for its high degree of endemism—up to 35.35%—which contributes to the floristic uniqueness and the irreplaceability of CA. Tajikistan, encompassing the most dominant area of the CA mountains, has the highest species density (3.19/100 km^2^) and endemism (11.12%) among the five countries. Neighboring countries such as Tajikistan and Uzbekistan, and Kazakhstan and Kyrgyzstan share more species in common, while Turkmenistan has less species overlap with the other four countries. Trends in endemic and total taxa are consistent. This comprehensive inventory is novel, revealing CA’s plant diversity in two dimensions and providing a solid foundation for subsequent research that will be beneficial to the transboundary conservation and sustainable use of plant resources in CA.

## 1. Introduction

Central Asia (CA), sometimes referred to as Middle Asia [1], is most commonly defined by the five “stans” of Kazakhstan, Kyrgyzstan, Tajikistan, Turkmenistan, and Uzbekistan (Figure S1). Geographically, CA spans nearly 4 million km^2^ and is located centrally within the world’s largest land mass, the Eurasian continent, serving as a pivotal bridge between Europe, the Near East, South Asia, and East Asia [2]. The region of CA is typically characterized by a blend of high mountain ranges, vast plains, expansive deserts, and fertile valleys, contributing to its unique and varied landscapes. There is mountainous terrain, such as the Tian Shan mountain range and the Pamir Plateau in the southeast, and lowlands in the northwest, centered on relatively flat land [3,4,5].

Simultaneously, CA is deeply landlocked and represents the largest arid region in the temperate and warm temperate zones of the Northern Hemisphere. The climate is largely arid to semi-arid, influenced significantly by westerly air circulation patterns [6]. Known as the “Water Tower” of CA, the presence of the Tian Shan mountain range creates an ecological barrier that blocks westerly wind circulation and affects precipitation distribution across the region [5,7,8]. The mountain ranges form a wet island amidst arid surroundings, crucially sustaining oasis stability, preserving water resources, and fostering diverse flora.

Such locations, as well as considerable variations in biogeographic, topographic, and climatic habitats, are favorable to high plant species diversity and a significant percentage of endemism in the flora.

Floristically, the CA is a part of the Irano–Turanian region and shows the greatest similarity with its western part, which stretches from the Anatolian Plateau to the Tian Shan and Pamir Mountains [9]. These two major mountain ranges, the Pamirs and the Tian Shan, comprise the Mountains of CA and are recognized as an important global biodiversity hotspot [10,11]. The CA mountains support around 7000 species of vascular plants, accounting for more than 75% of the total plant diversity in the region [12,13,14], and the vegetation types are mostly semi-desert and steppe ecosystems at both lower and higher altitudes, with a substantial variety of endemics [9].

Furthermore, CA has been hypothesized as the primary source and center of diversity for the current xerophytes found across Eurasia, the Mediterranean Basin, North Africa, and potentially South Africa [9]. The CA deserts harbor numerous plant species specifically adapted to arid conditions. These desert ecosystems are home to a variety of halophytes [15] and are centers of the origin and differentiation of ephemeral plants, containing more than 400 such species [16,17]. The unique adaptations of these species allow them to survive and even flourish in such harsh environments. 

Historically, modern botanical exploration of CA began in the 18th century, and a wealth of studies were conducted during the latter half of the 19th century [12,18,19]. During the 20th century, a large number of publications were produced that provided invaluable information about plant species diversity in the region [18,20,21]. A great contribution to our understanding of the flora was made by the research of Popov (1927) [22], Korovin (1962) [18], and Kamelin (1973) [12]. 

In the 21st century, the year 2015 was a momentous occasion for CA when the final volume (XI volume) of the *Conspectus Florae Asiae Mediae* was published by Khassanov (2015) [23]. It is an addendum to the previous volumes and can be regarded as a floristic checklist for CA. In the same year, the Korea National Arboretum developed a joint Korean-Central Asia project entitled the “Central Asia Green Road Project”, which includes a series of research projects and education programs aimed at CA biodiversity conservation. A book series devoted to the flora of the Tian Shan mountains was published with funding from the project [13]. Research on CA plants has intensified considerably since the initiation of the “Mapping Asian Plants (MAP) project” in November 2015, which is sponsored by the Biodiversity Committee of the Chinese Academy of Sciences. MAP established an online platform for big data on Asian plants, the core component of which is the compilation of a database of plant lists and distribution information [24]. As a critical initial step, Li et al. (2020) [4] reviewed historical botanical investigations, floristic works, and relevant botanical publications from the five CA countries to obtain basic data for the MAP project, which formed the basis of our study.

At the regional level, *Conspectus Florae Asiae Mediae* is the most noteworthy; it took 25 years to publish volumes I–X from 1968 to 1993. The most recent (XI) was published by Khassanov in 2015 and contains 9341 species of vascular plants belonging to 161 families and 1245 genera. A similar number of species (9346) were reported by Zhang et al. (2013) [16]. As research progresses, the number of species continues to increase. A total of 9520 species of higher plants were confirmed in 2020 [17], and we have made further revisions based on previous studies.

At the national level, the national Floras of five countries list 5631, 3576, 4445, 2607, and 4148 species of vascular plants in Kazakhstan [25], Kyrgyzstan [26], Tajikistan [27], Turkmenistan [28,29,30], and Uzbekistan [31]. These national Floras were published 33–92 years ago and are largely out of date with respect to species number, nomenclature, and geography. The updated vascular plant checklists in Kazakhstan [32], Kyrgyzstan [33], and Turkmenistan [21] were revised to 5658, 3927, and 2800, respectively. Currently, *Flora of Uzbekistan* includes more than 4400 species [34]. But this number is not the end because the launch of the international project “Flora of Uzbekistan” in 2016 [35] it has led to relevant investigations and has become an option for revision understudied taxa [36]. The first six volumes of the new edition of *Flora of Uzbekistan* containing treatments of 20 families with 184 genera and 820 species (about 20% of flora) have been published to date [37,38,39,40,41,42]. 

M. Nobis and A. Nowak, in collaboration with many Polish botanists, inherited the best traditions of European botanists who made a huge contribution to the knowledge of the flora of CA. Moreover, their research activities for more than twenty years have made a worthy contribution to the knowledge about the vegetation and rare and endangered plant species in the mountains of CA, a global biodiversity hotspot [43,44,45,46].

CA is a region with a distinct and diverse flora, as evidenced by its species richness, high level of endemism, taxonomic distinctiveness, unusual evolutionary history, and the global rarity of its major habitat types. However, these features often extend across national borders, adding to the multi-national and multi-ethnic dynamics of the area, which hinder integrated research on the flora of CA. Some studies are currently focused on the national scale, unfortunately, information about the region in its entirety remains scarce. This gap in knowledge is critical, given the region’s unique environmental conditions and the potential threats posed by climate change, habitat degradation, and human activities. Therefore, we have attempted in this study to reveal the status of vascular plant diversity in CA as follows: (1) by elucidating the plant diversity in both regional and national dimensions; (2) by compiling the basic information, including number of species, dominant families and genera, and endemic species according to our updated checklists; (3) by comparing the characteristics in terms of species composition among the five CA countries. This study aims to enhance our understanding of CA’s plant resources and establish a scientific basis for their effective conservation and sustainable utilization. 

## 2. Materials and Methods

### 2.1. Data Compilation

The compilation of data has benefited from a large number of published national and regional botanical records, key floras, checklists, literature, herbaria, databases, and online datasets in Central Asia. The taxonomic revisions of certain families and genera were updated and critically proofread in light of more recent studies, with the updating of newly published species and taxonomic changes. The systematic orders and taxonomic circumscription of the families were organized based on the following classifications: lycophytes and ferns by PPG I (2016) [47], gymnosperms by Christenhusz et al. (2011) [48], and Angiosperms by APG IV (2016) [49]. The standardized plant scientific names and authorship of species, genera, and families follow the International Plant Names Index (IPNI, 2024) and Plants of the World Online (POWO, 2024). Unreliable names, which resulted in duplicates, invalid names, and orthographic variants, were removed from the results. To verify the distribution of each taxon, we systematically studied the flora of each CA country and examined occurrence records based on the Global Biodiversity Information Facility (GBIF, 2024). Additionally, we conducted a thorough examination of specimens housed in the main herbaria in CA, including AA, PPIU, KG, and KSPI in Kazakhstan; FRU in Kyrgyzstan; TAD and KHOR in Tajikistan; ASH in Turkmenistan; SAMDU and TASH in Uzbekistan; as well as LE and MW in Russia [50] (Thiers, 2024). Endemic taxa refer to taxa that are unique to a single region within CA and have no distribution elsewhere outside that region. The statistics were calculated with reference to the above distribution, including endemism restricted to one country to five countries. Extinct taxa were considered to be historically endemic, while introduced taxa were not considered to be endemic. Non-endemic taxa were eliminated by checking POWO (POWO, 2024), *Conspectus Florae Asiae Mediae* [23], and the national floras of five countries. The number and representative families and genera of endemic taxa in CA were also presented in this study.

### 2.2. Data Statistic Analysis

Data statistics and visualization were prepared using Microsoft Excel 2021 and R v. 4.1.3. Floristic similarity among the five countries in CA was determined using Sørensen’s Dissimilarity Index (βsor) and nonmetric multidimensional scaling (NMDS). GraphPad Prism 9.0.0 and Evenn [51] (Yang et al., 2024) were utilized for data visualization and picture plotting. 

## 3. Results

### 3.1. Regionwide Species Richness

CA supports a wide diversity of plants, comprising 9643 species (including the infraspecific taxa) belonging to 1198 genera and 139 families. Only 0.06% of all species are lycophytes, which are represented by two families, three genera, and six species; 0.66% of all species are ferns, which are represented by 14 families, 25 genera, and 64 species, subspecies; and 0.37% of all species are gymnosperms, which are represented by three families, six genera, and 36 species, subspecies. Angiosperms make up the vast majority of the flora in CA—up to 98.90%, including 9537 species and infraspecific taxa grouped in 1164 genera and 120 families. Within the angiosperms, there are 7944 species of dicots and 1593 species of monocots (Table S1). 

**Figure 1:**
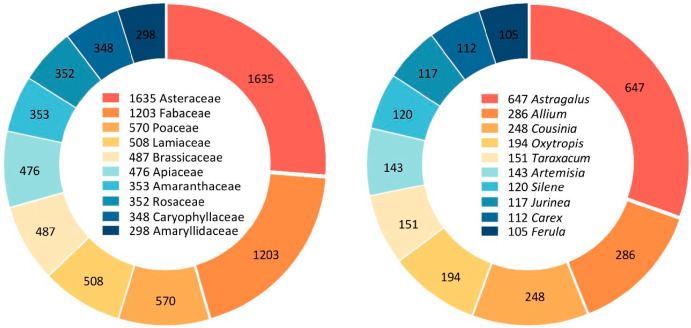
The top 10 dominant families and genera in the vascular flora of Central Asia.

Within the 139 recognized families, we consider those containing more than or equal to 100 taxa as large families, and a total of 19 large families are particularly rich in species and infraspecific taxa, totaling 7861 taxa and representing 81.52% of all species. Among them, the top 10 families were considered dominant families; they are, respectively, Asteraceae (1635), Fabaceae (1203), Poaceae (570), Lamiaceae (508), Brassicaceae (487), Apiaceae (476), Amaranthaceae (353), Rosaceae (352), Caryophyllaceae (348), and Amaryllidaceae (298), with a total of 6230 (64.61%) species, more than half of all species. Twenty-two families are represented by only one species in each. In terms of representative genera, we regarded genera with 100 or more taxa as large genera, and a total of 10 large genera coincided with the top 10 dominant genera, with a total of 2123 taxa, accounting for 22.02% of all species. They are as follows: *Astragalus* (647), *Allium* (286), *Cousinia* (248), *Oxytropis* (194), *Taraxacum* (151), *Artemisia* (143), *Silene* (120), *Jurinea* (117), *Carex* (112), and *Ferula* (105) (Figure 1). In addition, there are 477 monospecific genera that are represented by only one species each.

A large anthology of 3409 taxa are grouped in 66 families and 414 genera and are restricted in their distribution to this region. Endemics within this group correspond to 35.35% of the total flora. The most represented families and genera involved in the endemic taxa of CA are the Asteraceae (716) and *Astragalus* (364), which are the same as the dominant families and genera throughout CA. There are no endemic plant families in CA. The three genera *Cousinia* Cass. (199), *Allium* L. (154), and *Oxytropis* DC. (118) have a relatively large number of endemic species, all with more than 100 taxa. The endemic taxa in CA also include 1839 taxa from a single country, 853 taxa from two countries, 468 taxa from three countries, 212 taxa from four countries, and 37 taxa from all five countries (Figure S2).

### 3.2. Nationwide Species Richness

**Figure 2:**
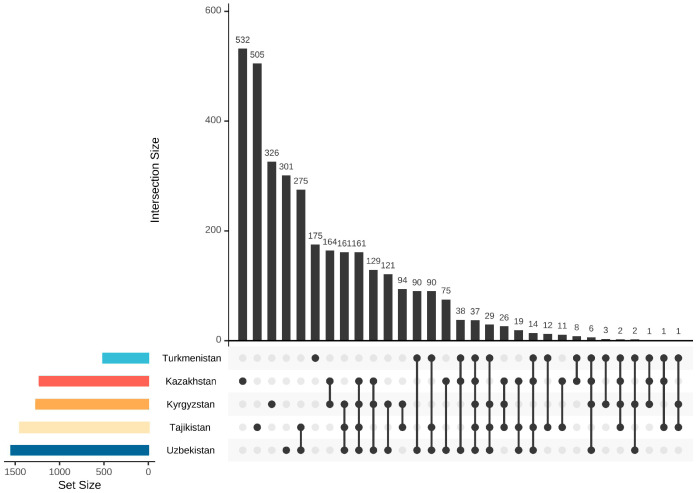
Similarity and endemism analysis of all the species in five countries. Intersection size: the number of shared endemic species.

**Figure 3:**
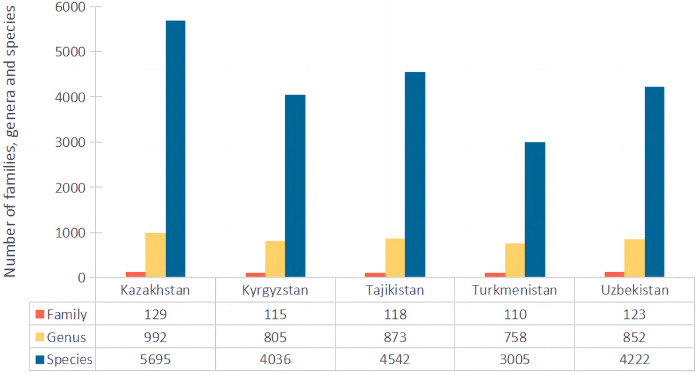
Number of families, genera, and species for five countries.

Within the CA, at the national level, considerable variation in species richness and endemism was observed. Kazakhstan (2,724,900 km^2^) stands out as the largest country and the most species-rich region, with 5695 species, 532 of which are endemic. Tajikistan takes second place with 4542 species, of which 505 are endemic, followed by Uzbekistan with 4222 species, of which 301 are endemic. Kyrgyzstan, with 4036 species, ranks fourth in terms of total species richness and, with 326 endemic species, ranks third in terms of endemism. Turkmenistan is in the last place with 3005 species, of which 175 are endemic (Figure 2 and Figure 3). In terms of the proportion of endemic taxa to total taxa, 11.12% of the vascular plant is restricted to Tajikistan, whereas only 5.82% of the flora of Turkmenistan is endemic. Similar percentages of endemism are shown in Kazakhstan, Kyrgyzstan, and Uzbekistan, with 9.34%, 8.08%, and 7.13%, respectively. 

**Figure 4:**
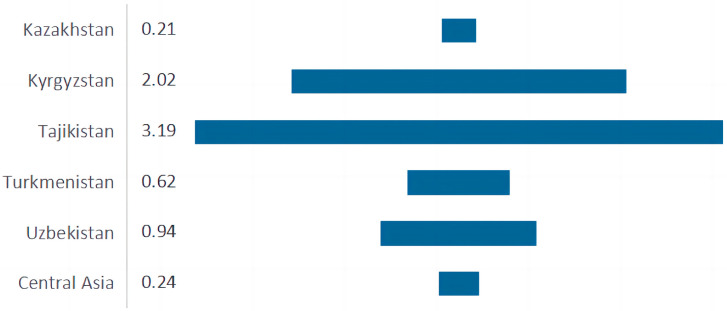
Species density represented by the number of species /100 km^2^.

Species densities per hundred km^2^ were computed for each country based on its size and the number of species and infraspecific taxa. Overall, CA encompasses a vast area with a regional species density (0.24) that is a little higher than that of Kazakhstan (0.21) but much lower than that of the other four countries. As shown in Figure 4, Tajikistan is obviously a hotspot for species richness, with the highest species density (3.19), holding more than 15 times the species than Kazakhstan, despite being the smallest country in terms of area (142,550 km^2^), which is one-nineteenth the size of Kazakhstan.

**Figure 5:**
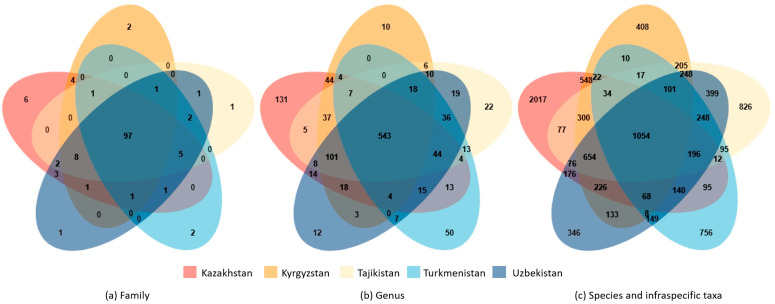
Venn diagrams showing shared families, genera, and species among five countries. The number at each intersection part indicated the number of shared families, genera, and species.

The floras of five countries exhibit some degree of similarity in the composition of families, genera, and species; with 97 families (69.78%), 543 genera (45.33%), and 1054 species (10.93%) shared by all countries (Figure 5).

As for the dominant families in their respective regions, the order of the first three families is consistent from the CA to CA countries. The Asteraceae is the most diverse family among CA countries; followed by the Fabaceae and then Poaceae (Figure 1). A total of eight dominant families are shared by all countries, and they are the Asteraceae, Fabaceae, Poaceae, Lamiaceae, Brassicaceae, Apiaceae, Amaranthaceae, and Caryophyllaceae. The dominant family Boraginaceae is shared by four countries except Kazakhstan; and the Rosaceae is shared by Kazakhstan, Kyrgyzstan, and Tajikistan. The Ranunculaceae is a dominant family unique to Kazakhstan and distinct from other countries, and the Polygonaceae is a dominant family in Turkmenistan but not in other countries, as is the Amaryllidaceae in Uzbekistan.

As for the dominant genera in their respective regions, the variations are significant. *Astragalus* (Fabaceae) is well represented in CA and across CA countries. Only four dominant genera are shared by all countries, and they are *Astragalus*, *Allium*, *Artemisia*, and *Silene*. Furthermore, *Oxytropis* is dominant in four countries except for Turkmenistan, as is *Cousinia* in Kazakhstan.

**Figure 6:**
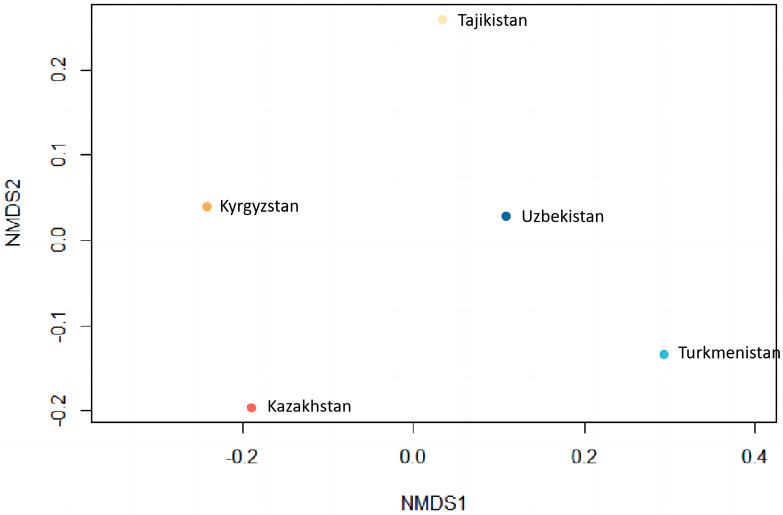
Floristic similarity among the five countries, based on nonmetric multidimensional scaling (NMDS).

Of the 9643 taxa in CA, there are 1054 taxa, which occur in all five countries considered here (Figure 5). Neighboring countries have lower Sørensen Dissimilarity Index values, indicating more species in common, such as Tajikistan and Uzbekistan (0.320858, 2976) (Table 1). Turkmenistan has a relatively higher Sørensen Dissimilarity Index than other countries, especially Kyrgyzstan, which has the lowest number of common species (0.626758, 1314). The floristic similarity among the five countries can also be gauged using a nonmetric multidimensional scaling (NMDS). Tajikistan shares more species with Uzbekistan than with any other area, and Kazakhstan has the most commonality with Kyrgyzstan. The point of Turkmenistan is distant from all other points, and thus, the flora of Turkmenistan shares fewer similarities with the other four countries (Figure 6).

Trends in endemic and total taxa are consistent. Tajikistan and Uzbekistan exhibit a high level of similarity, with as many as 275 endemic species shared. Kazakhstan and Kyrgyzstan have up to 164 co-occurring endemic species. However, fewer endemic species (three species) are shared between Kyrgyzstan and Turkmenistan (Figure 2).

## 4. Discussion

More than 50 years have passed since the last analysis of the flora of CA, carried out by R. Kamelin (1973) [12]. The accumulation of a huge amount of new plant taxa information and the digitization of a significant part of it allows us to analyze it to reveal the biodiversity status of vascular plants in CA with the level of endemism. The dataset we have accumulated allows us to carry this analysis out at the regional (CA) and national (for CA countries) levels.

Our findings support the view that plant taxa in CA are both rich and unique. As for the species-area ratio—the number of species per unit area—this measure reflects the mean species richness and serves as a direct indicator of the level of biodiversity in each region. The CA sustains 0.24 taxa/100 km^2^; among this, Tajikistan (3.19) and Kyrgyzstan (2.02) support more species than the other regions. This can be attributed to the fact that Tajikistan and Kyrgyzstan are typically mountainous regions. Tajikistan is located almost entirely within the Pamir-Alay mountain system, and more than half of the country is above 3000 m [43]. Additionally, most parts of Kyrgyzstan are occupied by high mountains and their foothills, with the Tian Shan (to the north, north-west, and east) and Pamir-Alay (to the south-west) being the prominent mountain systems [52]. The diverse eco-geographical conditions in mountainous regions create a typical vertical zonation, resulting in a rich plant diversity. As a globally important biodiversity hotspot, the mountains of CA, dominated by the Tian Shan and Pamir-Alay mountain systems, sustain about 7000 species of vascular plants, accounting for the vast majority of plant diversity in this region [13,14].

In other subregions of Asia, such as Northeast Asia, the species density is 0.25 taxa/100 km^2^ [53]. Numerically, although CA has no advantage in terms of species-area ratio, it is still a carrier of a rich gene pool of plant diversity, providing a wide range of ecosystem products and services for regional sustainability. It is a center of origin and diversity for a number of important taxa and some economically valuable plants such as medicinal plants like *Ferula* [54]; ornamental plants like *Tulipa* [55], *Eremurus* [56], and *Juno-Irises* [57]; and economically valuable plants like wild apples [58]. According to the available data from 1930 to the present, more than half of CA plants produce alkaloids [59].

Stress-resistant plant resources with excellent characteristics such as salt tolerance, drought tolerance, and high light efficiency have significant potential for the future bio-industry. There are also certain ephemeral plants in deserts that can utilize snowmelt water in early spring to complete their life histories rapidly, e.g., *Carex pachytylis*, *Poa bulbosa,* and *Ranunculus pinnatisectus*, which provide material for ecological adaptation research [16]. The arid zone of CA is home to many relict tertiary species of ancient Mediterranean origin, such as the *Nitraria* and *Caragana*, which are important materials for geological history research [60]. The abundance of endemic species in a region is often used as evidence supporting the designation of that area as a diversity hotspot [61]. The undeniable value of CA is in its high degree of endemism at both national and regional levels [62], with up to 20% of the flora restricted to the region [16]. Since the completion of our study, this percentage may be higher, reaching 35.35%. Generally, we believe that larger areas have more range-restricted species [63], such as Kazakhstan being the ninth largest country globally and the largest country in CA, with the largest number of endemic taxa. Furthermore, higher sites host more native and endemic plant taxa than adjacent lowlands [64], with endemic species richness being higher in southeastern CA, that is, Tajikistan (11.12%) and Kyrgyzstan (8.08%). These significant figures indicate the irreplaceability of the floras in CA.

Additionally, Important Plant Areas (IPA) are key sites for rich biodiversity, rare and threatened plant species, and socio-economically valuable plant species [65]. IPAs also harbor a substantial number of endemic plants, contributing further to their ecological value and conservation significance. Fergana Valley is one of these regions, located in the eastern region of Uzbekistan (7.13%). The valley represents an ancient, isolated ecoregion with unique flora and fauna, much of which is endemic to the valley [66] and has the highest level of endemic biodiversity in CA [67,68].

The number of endemic plants in the regions neighboring CA varies, with China exhibiting the highest number of endemic species at 14939 [69]. Xinjiang of China borders CA and possesses 251 endemic plant species (Li et al., unpublished data). There are over 2700 endemic taxa in Russia [70], and Mongolia has 102 taxa [71]. In comparison, CA remains highly endemic, with more than 3000 endemic plant species. Previous studies found that a group of 1243 endemic and 257 sub-endemic vascular plant species are present in Tajikistan [44], while Kazakhstan has 451 endemic and 341 sub-endemic taxa [72], Kyrgyzstan has 393 endemic taxa [33], and Uzbekistan has 313 endemic taxa [34]. Differences in the number of estimated species vary, as we defined the distribution of endemic species to be strictly “endemic” rather than “sub-endemic” [44]. On the other hand, we also found that more than 100 species were omitted from the count of endemics in Kazakhstan [72].

Another important result was that, despite the high degree of endemics, the number of widely distributed taxa in CA is limited, with 1054 taxa occurring in all five countries, accounting for 10.93% of the total. It is quite possible that the latitudinal span of the entire region leads to north-south variances, as well as differences in eco-geographical environment, which in turn influences the differences in species composition. Regionally, a high proportion of endemism is accompanied by a decline in commonality. On the other hand, within CA countries, the proportion of shared species ranges from 18.50% to 35.06%, which also represents a significant portion. From the perspective of plant taxonomy, this could also be owing to the relatively fine-grained division of species in CA, as there is a high proportion of commonality between families (69.78%) and genera (45.33%).

Despite this work being novel for CA as a whole, the large quantity of data presented in this inventory provide a solid beginning point for a thorough investigation of endemic, endangered, and other key groups of species throughout CA and CA countries. More botanical surveys and further analyses are needed to better describe CA’s floristic diversity. A key strategy is to strengthen intra- and interregional cooperation. Comprehensive conservation plans should be regional, complementing national plans, as CA’s mountain ranges, deserts, and vegetation formations usually stretch across country borders. This is particularly true for threatened species, where assessments at the national level alone can be misleading, and large numbers of taxa with transnational distributions, such as tulips [73,74], need to be studied at larger scales, especially in the face of climate change. 

At a time when global plant diversity data are being utilized to investigate biodiversity conservation, conservation assessments only at the national scale, as well as the lack of transboundary conservation, limits its research and effective conservation in CA, which should be addressed regionally. Under the key 2020 Aichi Biodiversity Targets, the current status of plant conservation in the five CA countries is unfortunately incompatible with the above targets and, therefore, the conservation of regional biodiversity has been recognized as an important objective [75]. Furthermore, combined regional and national research is beneficial to all perspectives and promotes cooperation among countries. International collaborative research should thus be fostered to the greatest extent feasible. In addition to the practical value of collaborative research, such research can contribute to better relations between countries and demonstrate the advantages of collaboration, which can subsequently be applied to other more challenging fields [76,77].

CA is characterized by a blend of high mountain ranges, vast plains, expansive deserts, and fertile valleys, contributing to its unique and varied landscapes. However, the region faces significant environmental challenges, especially in an era of intense and rapid climatic changes. Human activities such as deforestation and overgrazing have collectively contributed to the degradation of its ecosystems and caused a decline in plant diversity across the region, marking the region as “nature imperiled” [78]. Volis (2023) [75] provides a detailed conservation methodology and conservation planning useful for CA countries, with an ecoregional basis and the highest priority given to the most endangered endemic species. In addition, conservation measures should be upgraded in some key geographical areas, such as the IPA of Fergana Valley. 

Overall, successfully adopting and implementing all these strategies in CA requires enhanced coordination in developing and executing conservation plans among scientists and governments from the five CA countries.

## Figures and Tables

**Table 1:** Number of plant species in each country (diagonal in bold) in Central Asia. Above the diagonal: number of shared taxa between two countries. Below the diagonal: Sørensen dissimilarity Index, values between 0 and 1.

	Kazakhstan	Kyrgyzstan	Tajikistan	Turkmenistan	Uzbekistan
Kazakhstan	**5695**	2906	2403	1622	2591
Kyrgyzstan	0.402734	**4036**	2613	1314	2492
Tajikistan	0.530527	0.390767	**4542**	1757	2976
Turkmenistan	0.627126	0.626758	0.534385	**3005**	1964
Uzbekistan	0.477463	0.396464	0.320858	0.456483	**4222**

## Data Availability

The data presented in this study are available from the corresponding author upon reasonable request.

## Supplementary Materials

The following supporting information can be downloaded at: https://www.mdpi.com/article/10.3390/plants13162275/s1, Figure S1: Five countries in Central Asia, by Family, Genus, Species and Area; Figure S2: The endemic taxa in Central Asia from one country to five countries; Table S1: Taxonomic distribution of the vascular flora of Central Asia.
