# Supplementary material for: Regionwide and Nationwide Floristic Richness Reveal Vascular Plant Diversity in Central Asia
Source: Plants (Basel). 2024 Aug 15;13(16):2275. doi: 10.3390/plants13162275 (PMC11360335; doi:10.3390/plants13162275)
Supplement: Supplementary file 1 [file plants-13-02275-s001.zip › plants-3114920-supplementary.pdf]

Supplementary Materials:

Table S1. Taxonomic distribution of the vascular flora of Central Asia

	**Families**	**Genera**	**Species and infraspecific taxa**
Lycophytes	2	3	6
Ferns	14	25	64
Gymnosperm	3	6	36
Angiosperms:	120	1164	9537
Monocots	25	213	1593
Dicots	95	951	7944
**Total**	**139**	**1198**	**9643**

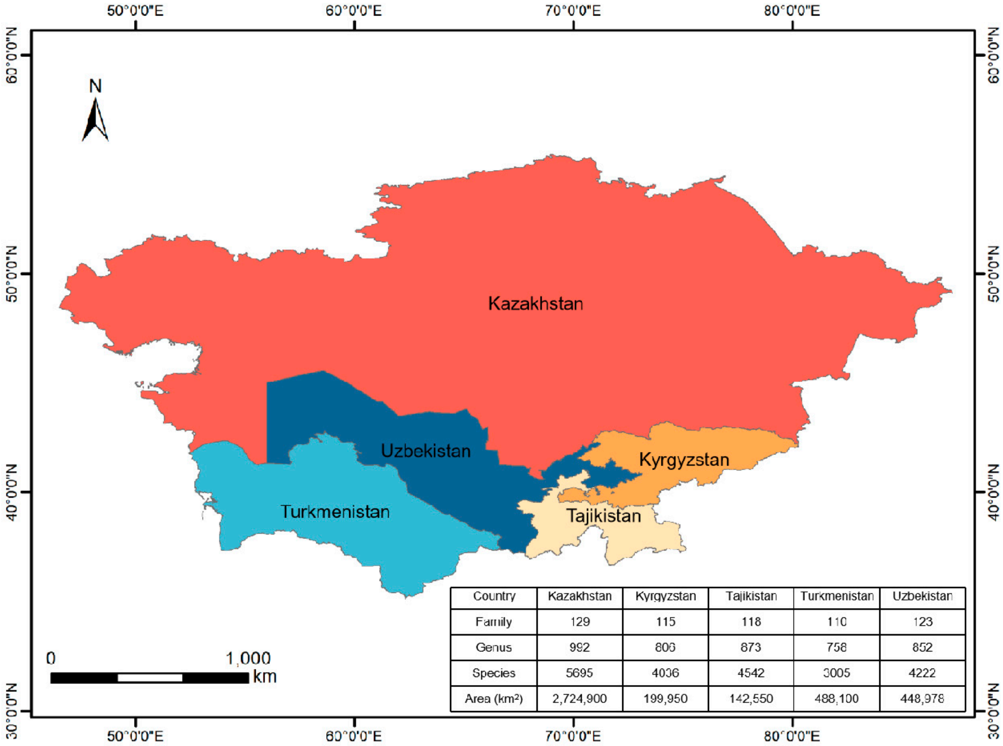

Country	Kazakhstan	Kyrgyzstan	Tajikistan	Turkmenistan	Uzbekistan
Family	129	115	118	110	123
Genus	992	806	873	758	852
Species	5695	4036	4542	3005	4222
Area (km²)	2,724,900	199,950	142,550	488,100	448,978

Figure S1. Five countries in Central Asia, by Family, Genus, Species and Area.

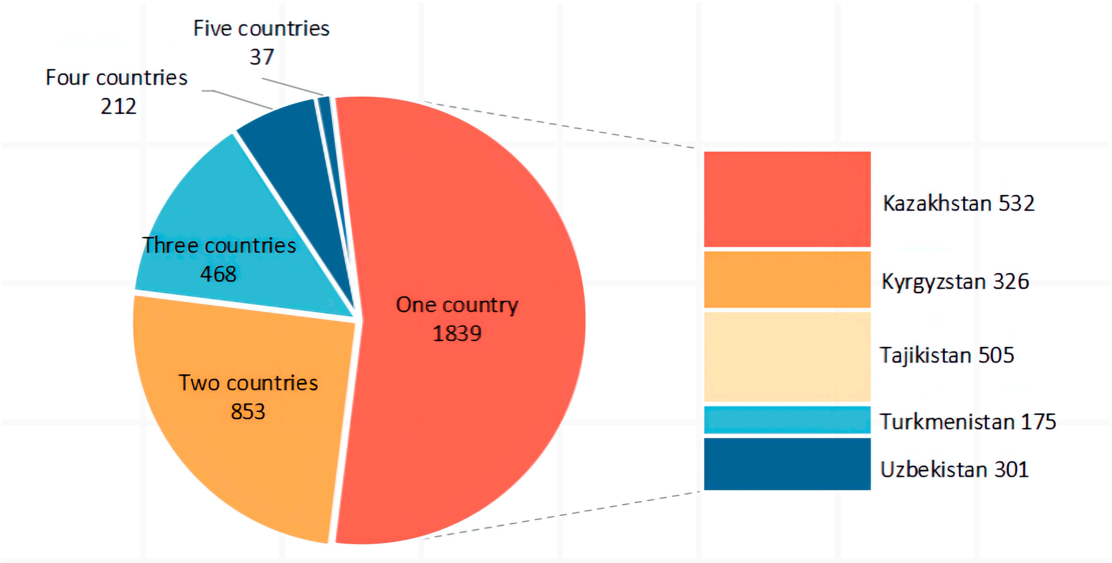

Figure S2. The endemic taxa in Central Asia from one country to five countries.
